# Supplementary material for: Assessment of the Impact on 20 Pelagic Fish Species by the Taiwanese Small-Scale Longline Fishery in the Western North Pacific Using Ecological Risk Assessment
Source: Animals (Basel). 2022 Aug 19;12(16):2124. doi: 10.3390/ani12162124 (PMC9404817; doi:10.3390/ani12162124)
Supplement: Supplementary file 1 [file animals-12-02124-s001.zip › animals-1830045-supplementary.pdf]

Supplemental Table S1. Age and growth parameters of the 20 pelagic species in the Northwest Pacific Ocean.

Scientific Name	L_{max}(cm) EFL	L_{max}(cm) LJFL	L_{max}(cm) FL	L_{max}(cm) TL	L_{∞}(cm)	k(yr^{-1})	t_0	A_{max}(yr)	References
Alopias pelagicus				365.18	382.94	0.085	-7.67	27.57	[54]
A. superciliosus				422.00	422.00	0.092	-4.21	28.35	[55]
Carcharhinus brevipinna				274.00	288.20	0.151	-1.99	17.85	[56]
C. falciformis				256.00	332.00	0.084	-2.76	32.99	[57]
C. longimanus				268.00	323.80	0.011	-0.37	12.30	[50]
C. obscurus				364.00	415.70	0.056	-3.42	50.08	[58]
C. plumbeus				210.00	210.00	0.170	-2.30	15.32	[59]
Isurus oxyrinchus				375.00	413.80	0.050	—	40.04	[6]**
Sphyrna lewini				324.00	319.72	0.249	-0.41	11.62	[43]
S. zygaena				324.00	375.20	0.111	-1.31	25.73	[60]
Prionace glauca				323.00	322.70	0.161	-1.33	17.24	[61]
Makaira nigricans	276.00				421.83	0.009	-1.76	14.00	[38]***
Istiompax indica	324.50*	368.20			396.57	0.094	-1.83	11.00	[39]
Istiophorus platypterus	207.16*	239.00			343.80	0.011	-0.47	12.00	[15]***
Xiphias gladius	265.06 *	290.00			300.66	0.040	-0.75	12.00	[37]***
Kajikia audax	191.00	220.90*			277.40	0.170	0.22	6.00	[40]***
Thunnus alalunga			101.00		103.50	0.340	-0.53	10.00	[14]
T. albacares			164.60		175.00	0.392	0.00	7.65	[36]
T. obesus			183.10		203.80	0.212	-0.91	13.22	[12]
T. orientalis			252.00		366.68	0.086	-0.93	13.00	[41]

*: Converting from different measurements using conversion factors. **: two-parameter VBGF, ***: Richard growth function.

L_{max}: maximum observed length, L_{∞}: asymptotic length, k: growth coefficient, t_0: age at length 0, A_{max}: longevity.

Supplemental Table S2. Reproductive parameters of 11 pelagic shark species in the Northwest Pacific Ocean.

Scientific name	R	L_b (cm)	L_m (cm)	A_m (yr)	f	G_p (month)	R_c (yr)	References
Alopias pelagicus	ov	174	287	8.6	2	12	1	[2]
A. superciliosus	ov	148.7	336.58	12.85	2	12	1	[62]
Carcharhinus brevipinna	v	67.5	222.5	7.8	8.5	11	2	[56]
C. falciformis	v	69.5	215	9.7	9	12	2	[57]
C. longimanus	v	64	194.7	8.23	10	12	2	[50]
C. obscurus	v	101	281	16.4	11	13	2	[58]
C. plumbeus	v	62.5	172.5	7.85	7.5	11	2	[63]
Isurus oxyrinchus	ov	74	278	20	11.1	24	3	[61]
Sphyrna lewini	v	48.5	230	4.7	25.8	10	2	[64]
S. zygaena	v	55	259.4	11	30	10	2	[65]
Prionace glauca	v	45	189	4.2	29	10	2	[66]

R: reproductive type (ov: aplacental viviparity, v: viviparity), L_b: size at birth, L_m: size at maturity, A_m: age at maturity, f: littler size, G_p: gestation period, R_c: reproductive cycle, sex ratio of embryos (F/(F+M)) was set as 0.5.

Supplemental Table S3. Reproductive parameters of 5 billfish and 4 tuna species in the Northwest Pacific Ocean.

Scientific name	R	L_m (cm) EFL	L_m (cm) LJFL	L_m (cm) FL	A_m (yr)	F (million)	References
Makaira nigricans	o	180.56			7.36	6.94±1.12	[67]
Istiompax indica	o	194.90	223.00		6.97	1.52±0.2	[68]
Istiophorus platypterus	o		166.38		5.00	1.299±0.365	[15]
Xiphias gladius	o		168.20		5.00		[69]
Kajikia audax	o	180.78			4.80	4.38±1.67	[70]
Thunnus alalunga	o			83.00	4.50	0.94±0.43	[71]
T. albacares	o			107.77	2.40	2.71	[72]
T. obesus	o			99.70	2.26		[73]
T. orientalis	o			190.00	8.00	5.8-25.2	[74]

R: reproductive type (o: oviparous), L_m: size at maturity, A_m: age at maturity, F: fecundity.

References:

54. Liu, K.M.; Chen, C.T.; Liao, T.H.; Joung, S.J. Age, growth, and reproduction of the pelagic thresher shark, *Alopias pelagicus*, in the northwestern Pacific. *Copeia* **1999**, *1999*, 68–74. https://doi.org/10.2307/1447386.
55. Liu, K.M.; Chiang, P.J.; Chen, C.T. Age and growth estimates of the bigeye thresher, *Alopias superciliosus*, in northwestern Taiwan waters. *Fish. Bull.* **1998**, *96*, 262–271.
56. Joung, S.J.; Liao, Y.Y.; Liu, K.M.; Chen, C.T.; Leu, L.C. Age, growth, and reproduction of the spinner shark, *Carcharhinus brevipinna*, in the northeastern waters of Taiwan. *Zool. Stud.* **2005**, *44*, 102–110.
57. Joung, S.J.; Chen, C.T.; Lee, H.H.; Liu, K.M. Age, growth, and reproduction of the silky sharks *Carcharhinus falciformis* in northeastern Taiwan waters. *Fish. Res.* **2008**, *90*, 78–85. https://doi.org/10.1016/j.fishres.2007.09.025.
58. Joung, S.J.; Chen, J.H.; Chin, C.P.; Liu, K.M. Age and growth of the dusky shark, *Carcharhinus obscurus*, in the Northwest Pacific Ocean. *Terr. Atmos. Oceanic Sci*. **2015**, *26*, 153–160. https://doi.org/10.3319/TAO.2014.10.15.01(Oc).
59. Joung, S.J.; Liao, Y.Y.; Chen, C.T. Age and growth of sandbar shark, *Carcharhinus plumbeus*, in northeastern Taiwan waters. *Fish. Res.* **2004**, *70*, 83–96. https://doi:10.1016/j.fishres.2004.06.018.
60. Chou, Y.T. Studies on Age and growth of smooth hammerhead, *Sphyrna zygaena* in northeastern Taiwan waters. Master's Thesis, National Taiwan Ocean University, Keelung, Taiwan, 2004; p. 66.
61. Huang, C.C. Age and growth of the blue shark, *Prionace glauca*, in the northwestern Pacific Ocean. Master's Thesis, National Taiwan Ocean University, Keelung, Taiwan, 2006; p. 76.
62. Chen, C.T.; Liu, K.M.; Chang, Y.C. Reproductive biology of the bigeye thresher shark, *Alopias superciliosus* (Lowe, 1839) (Chondrichthyes: Alopiidae) in the northwestern Pacific. *Ichthyol. Res.* **1997**, *44*, 227–235. https://doi.org/10.1007/BF02678702.
63. Joung, S.J.; Chen, C.T. Reproduction in the sandbar shark, *Carcharhinus plumbeus*, in the waters off northeastern Taiwan. *Copeia* **1995**, *1995*, 659–665. https://doi.org/10.2307/1446762.
64. Chen, C.T.; Leu, T.C.; Joung, S.J. Notes on reproduction in the scalloped hammerhead, *Sphyrna lewini*, in northeastern Taiwan waters. *Fish. Bull*. **1988**, *86*, 389–393.
65. Liu, S.C. Reproductive Biology of smooth hammerhead, *Sphyrna zygaena* in northeastern Taiwan waters. Master's Thesis, National Taiwan Ocean University, Keelung, Taiwan, 2002; p. 84.
66. Wu, T.Y. Reproductive biology of blue shark, *Prionace glauca* in the northwestern Pacific Ocean. Master's Thesis, National Taiwan Ocean University, Keelung, Taiwan, 2003; p. 109.
67. Sun, C.L.; Chang, Y.J.; Tszeng, C.C.; Yeh, S.Z.; Su, N.J. Reproductive biology of blue marlin (*Makaira nigricans*) in the western Pacific Ocean. *Fish. Bull*. **2009**, *107*, 420–432.
68. Liu, T.Y. Reproductive biology of black marlin, *Makaira indica*, in the waters off Taiwan and South China Sea. Master's Thesis, National Taiwan University, Taipei, Taiwan, 2007; p. 117.
69. Wang, S.P.; Sun, C.L.; Yeh, S.Z. Sex ratios and sexual maturity of swordfish (*Xiphias gladius* L.) in the waters of Taiwan. *Zool. Stud.* **2003**, *42*, 529–539.
70. Chang, H.Y. Reproductive biology of striped marlin, *Kajikia audax*, in the waters off Taiwan. Master's Thesis, National Taiwan University, Taipei, Taiwan, 2011; p. 108.

71. Chen, K.S.; Crone, P.R.; Hsu, C.C. Reproductive biology of albacore *Thunnus alalunga*. *J. Fish. Biol*. **2010**, *77*, 119–136. https://doi.org/10.1111/j.1095-8649.2010.02662.x.
72. Wang, W.J. Reproductive biology of yellowfin tuna *Thunnus albacares* in the western Pacific Ocean. Master's Thesis, National Taiwan University, Taipei, Taiwan, 2005; p. 146.
73. Ju, S.L. Reproductive biology of bigeye tuna *Thunnus obesus* in the western Pacific Ocean. Master's Thesis, National Taiwan University, Taipei, Taiwan, 1998; p. 67.
74. Chen, K.S.; Crone, P.R.; Hsu, C.C. Reproductive biology of female Pacific bluefin tuna *Thunnus orientalis* from south-western North Pacific Ocean. *Fish. Sci.* **2006**, *72*, 985–994
